# Association of methylenetetrahydrofolate reductase (MTHFR) rs1801133 (677C>T) gene polymorphism with ischemic stroke risk in different populations: An updated meta-analysis

**DOI:** 10.3389/fgene.2022.1021423

**Published:** 2023-01-04

**Authors:** Lili Zhao, Tao Li, Meijuan Dang, Ye Li, Hong Fan, Qian Hao, Dingli Song, Jialiang Lu, Ziwei Lu, Yating Jian, Heying Wang, Xiaoya Wang, Yulun Wu, Guilian Zhang

**Affiliations:** ^1^ Department of Neurology, The Second Affiliated Hospital of Xi’an Jiaotong University, Xi’an, China; ^2^ Department of Oncology, The Second Affiliated Hospital of Xi’an Jiaotong University, Xi’an, China; ^3^ Department of Thoracic Surgery, The First Affiliated Hospital of Xi’an Jiaotong University, Xi’an, China

**Keywords:** polymorphism, ischemic stroke, meta-analysis, risk, MTHFR rs1801133 (677C>T)

## Abstract

**Background:** Recently, increasing evidence has implicated methylenetetrahydrofolate reductase (MTHFR) gene mutation as a risk factor for ischemic stroke (IS) in the general population. However, studies have been inconclusive and lack evidence on specific populations. We aim to determine whether the rs1801133 (NC_000001.11 (MTHFR):g. 677C>T (p.Ala222Val) variant, we termed as MTHFR rs1801133 (677 C>T), is linked to an increased risk of IS in different age groups and ancestry groups.

**Methods:** The literature relevant to our study was found by searching the PubMed, Cochrane Library, Web of Science, EMBASE, and CNKI databases. A random effect model analysis was used to calculate the pooled odds ratio (OR) and 95% confidence interval (CI) to evaluate any possible association. We conducted a subgroup analysis based on the age and ancestry groups of the included populations.

**Results:** As of March 2022, 1,925 citations had been identified in electronic databases, of which 96 studies involving 34,814 subjects met our eligibility criteria. A strong link was found between IS and the MTHFR gene rs1801133 (677C>T) polymorphism in all genetic models [dominant genetic model (OR = 1.47; 95%CI = 1.33–1.61; *p* < 0.001), recessive genetic model (OR = 1.52; 95%CI = 1.36–1.71; *p* < 0.001), heterozygous model (OR = 1.36; 95%CI = 1.24–1.48; *p* < 0.001), homozygous model (OR = 1.82; 95%CI = 1.58–2.11; *p* < 0.001), and T allelic genetic model (OR = 1.37; 95%CI = 1.27–1.48; *p* < 0.001)]. Further subgroup analyses indicated that the MTHFR rs1801133 (677C>T) variant may increase the risk of IS in Asian, Hispanic, or Latin population, middle-aged, and elderly populations (*p* < 0.001).

**Conclusion:** Our results implied that mutation of the T allele of MTHFR rs1801133 (677C>T) could be a risk factor for IS. A significant association was found among Asian, Hispanic, or Latin population, middle-aged, and elderly people.

## 1 Introduction

**FIGURE 1:**
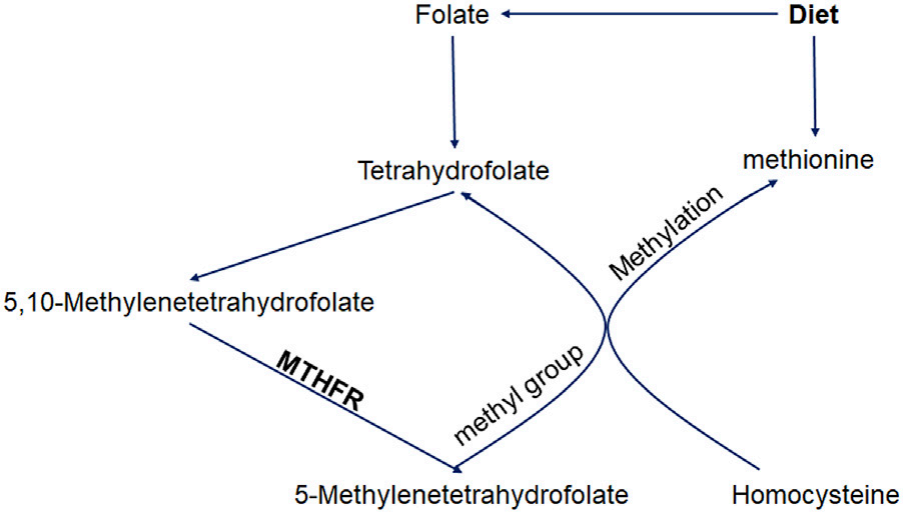
Roles of methylenetetrahydrofolate reductase in homocysteine metabolism.

Ischemic stroke (IS) is an acute neurological deficit caused by vascular occlusion. It is one of the leading causes of death and disability worldwide (Francis et al., 2007; Malik and Dichgans, 2018; Phipps and Cronin, 2020) and is caused by a combination of environmental and genetic factors (Black et al., 2015; Prabhakaran et al., 2015; Malik et al., 2019). Several pathophysiological mechanisms are involved in the development of this condition. Hyperhomocysteinemia is reported to be independently associated with the risk of stroke (Linnebank et al., 2012). The 5,10-methylenetetrahydrofolate reductase (MTHFR) locus is mapped to chromosome 1 (1p36.3) which encodes for the dimeric proteins of 70–77 kDa subunits (Goyette et al., 1994). Folate metabolism is largely controlled by MTHFR, which catalyzes the conversion of 5,10-methylenetetrahydrofolate to 5-methylenetetrahydrofolate. 5-Methylenetetrahydrofolate provides a methyl group in the methylation reaction that transforms homocysteine into methionine (Figure 1), as well as the DNA methylation process (Brattström et al., 1998). Thus, the MTHFR enzyme activity is important for homeostasis of the serum homocysteine level.

The previous study had demonstrated that approximately 40% of the intragenic coding CpG islands were hyper-methylated, which had a higher C>T mutation rate. Also, the amino acid sequence of a protein and individual phenotypes could be changed by C>T substitutions at the CpG contexts in the protein-coding regions (Youk et al., 2020). The rs1801133 variant (NC_000001.11 (MTHFR):g. 677C>T (p.Ala222Val), also named MTHFR rs1801133 (677C>T), is a common mutant in MTHFR. The replacement of C with T at nucleotide 677 results in converting alanine to valine amino acid residue in the enzyme (Sharp and Little, 2004). Missense mutations cause a 50%–60% decrease in enzyme activity in patients who have the homozygous variant (TT) (Rozen, 199 7), which contributes to hyperhomocysteinemia (Castro et al., 2004). In addition, reduction of the MTHFR enzymatic activity would cause deficiency of folate, which is also an independent risk factor of IS (Qin et al., 2020). Moreover, when folic acid o is inadequate, the removal of homocysteine would be affected, leading to hyperhomocysteinemia and forming a vicious cycle (Liew and Gupta, 2015). Thus, it is important to determine the association between MTHFR rs1801133 (677C>T) polymorphism and the risk of IS for primary and secondary prevention of IS.

Many researchers have examined the relationship between MTHFR rs1801133 (677C>T) polymorphism and IS risk. However, there have been no definitive conclusions because different populations were examined and inconsistent results were obtained (Herak et al., 2017; Jiménez-González et al., 2021; Huang et al., 2022). Two meta-analyses were performed separately in 2016 and 2019 that reported a correlation between MTHFR rs1801133 (677C>T) polymorphism and IS (Song et al., 2016; Chang et al., 2019). However, only 22 studies were included in Song et al. (2016). Since then, many studies have been conducted in different populations. Moreover, the meta-analysis by Song et al. focused on the general population and did not consider whether MTHFR rs1801133 (677C>T) polymorphism might have varying effects on the risk of IS in different populations. Furthermore, Guilin Chang et al.‘s study, published in 2019, included only nine studies on the elderly population and did not consider young and middle-aged IS patients (Chang et al., 2019). Therefore, past meta-analyses were updated to investigate whether MTHFR rs1801133 (677C>T) polymorphism and stroke risk are related across age and ancestry groups in this study.

## 2 Materials and methods

The study follows the Preferred Reporting Items for Systematic Reviews and Meta-Analyses guidelines (Moher et al., 2009).

### 2.1 Literature search

A systematic search of the PubMed, EMBASE, Cochrane Library, Web of Science, and CNKI databases for relevant observational studies published until 15 March, 2022, was undertaken independently by two reviewers (Zhao and Li). We used the following search terms to identify eligible studies: (“methylenetetrahydrofolate reductase” OR “MTHFR” OR “C677T” OR “rs1801133”), (“ischemic stroke” OR “cerebral infarction” OR “stroke”), AND (“single nucleotide polymorphism” OR “SNP” OR “genetic polymorphism” OR “mutation” OR “variation”). We reviewed the full text of each study when abstracts and titles were insufficient to make a final determination regarding study inclusion. The reference lists of included studies and existing reviews were screened to identify additional eligible studies. Any disagreements in the study selection process were resolved by a third person (Dang).

### 2.2 Selection criteria

We included the studies according to the following inclusion criteria: 1) the full text could be searched in electronic databases; 2) the studies were case-control or cohort studies examining MTHFR rs1801133 (677C>T) and stroke susceptibility; 3) the study population was limited to patients diagnosed with stroke for the first time; 4) the MTHFR rs1801133 (677C>T) genotype frequency was provided; and 5) articles were published in English or Chinese. The main exclusion criteria included the following: 1) the studies were duplicate articles or non-original research (letters, commentaries, editorials, reviews, and meta-analyses); 2) the studies were case reports or involved animal experiments; 3) the genotype frequency of MTHFR rs1801133 (677C>T) was not provided; and 4) the *p*-value of the Hardy–Weinberg equilibrium (HWE) test was <0.05.

### 2.3 Data extraction and quality assessment

A pre-designed extraction form was used to extract the data. The extracted data included the name of the first author, type of stroke, publication date, ancestry groups, sample size (case and control), study design, mean or median age of the population, HWE, and the Newcastle–Ottawa scale score. Disagreements regarding data extraction were resolved by discussions among the two investigators (Zhao and Li), and a third reviewer (Dang) was consulted if necessary. The HWE *p*-value was also calculated using the genotypic frequencies of MTHFR polymorphisms, and the threshold of HWE deviation was set at 0.05. The quality of eligible publications was assessed using the Newcastle–Ottawa scale (Stang, 2010), and studies with a score of 7–9 were considered to be of good quality.

### 2.4 Statistical analysis

We used five genetic comparison models, the T allelic model (T *vs*. C), dominant model (TT + TC *vs.* CC), recessive model (TT *vs*. CC + TC), heterozygous model (TC *vs.* CC), and homozygous model (TT *vs.* CC), to estimate the relationship between MTHFR rs1801133 (677C>T) polymorphism and stroke susceptibility by calculating the odds ratio (OR) and 95% confidence interval (CI). The I^2^ statistic was used to evaluate heterogeneity among genetic comparison models, and the pooled OR was estimated *via* the Mantel-Haenszel random effect model. Heterogeneity between studies is indicated by an I^2^ > 50%. Moreover, the Bonferroni method was utilized to adjust for multiple comparisons to control the false positive error rate. As we performed multiple comparisons in this meta-analysis for 45 times, the *p*-value which was less than 0.05/50 (0.001) indicated statistical significance after Bonferroni correction. To determine the possible causes of heterogeneity, the ancestry groups (Asian, European, African, Hispanic, or Latin American (HLA), and other and not reported ancestries (ONR)) and the study population (young: <18 years; middle-aged: 18–60 years; elderly: >60 years) were analyzed in subgroups. In addition, we examined the impact of a single study on the pooled OR by performing sensitivity analyses on different genetic comparison models. Egger’s test, Begg’s test, and funnel plots were used to evaluate the potential publication bias in our study (Peters et al., 2006). Stata 17.0 was used to perform the statistical analysis of all genetic comparison models.

## 3 Results

### 3.1 Literature search and characteristics of the included studies

**FIGURE 2:**
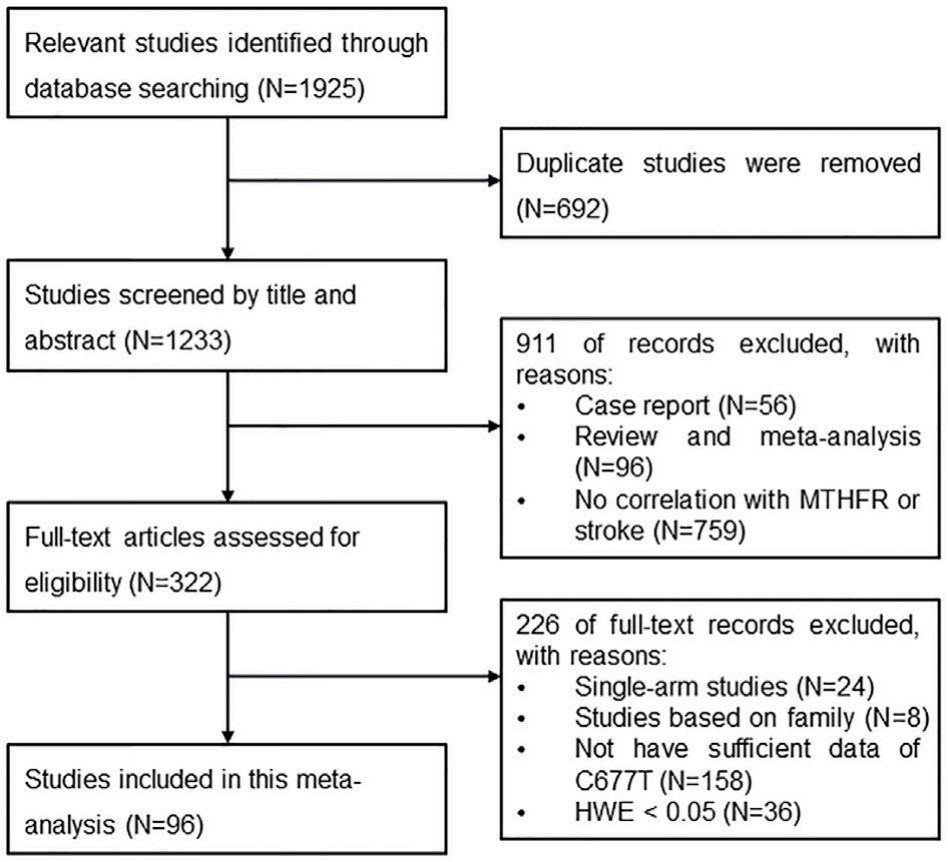
Flow chart of identification of eligible studies.

There were 1,925 articles initially identified after searching the databases. Among them, 692 articles were removed because of duplication, and 911 articles were removed after the titles and abstracts were screened. In total, 322 articles were further screened for eligibility by reviewing their full texts, and eventually, 96 articles that met the criteria were selected (Figure 2).

Of the 96 studies included, 95 were case-control studies, and one was a nested case-referent study. In total, 52 studies were conducted in the Asian population, 19 were in the European population, 1 was in the African population, 3 were in the Hispanic or Latin American population, and 21 were in other and not reported ancestry population. In total, 14 studies examined children, 27 examined middle-aged people, and 42 examined the elderly population. The other 13 studies examined the general population. The main features of the included studies are shown in Table 1.

**TABLE 1:** Characteristics of studies included in the meta-analysis.

Author	Year	Disease	Ancestry	Case			Control			Age	Design	HWE	NOS
				CC	CT	TT	CC	CT	TT				
Huang et al., (2022)	2022	IS	Asian	94	72	32	101	62	5	>18	Case-control	0.21	8
Salomi et al., (2021)	2021	IS	Asian	69	32	4	171	40	4	32.7±7.7	Case-control	0.36	8
Cernera et al., (2021)	2021	IS	European	64	140	76	138	206	85	8	Case-control	0.61	7
Jiménez-González et al., (2021)	2021	IS	HLA	35	105	38	60	83	40	34.1±5.4	Case-control	0.27	8
Mazdeh et al., (2021)	2021	IS	ONR	157	124	37	219	155	26	48±0.10	Case-control	0.84	7
Hou et al., (2018)	2018	IS	Asian	1063	793	138	1427	988	150	68.6±12.1	Case-control	0.22	9
Mao and Han, (2018)	2018	IS	Asian	73	189	27	82	100	16	68.42±13.26	Case-control	0.06	8
Li et al., (2017)	2017	IS	Asian	71	134	95	106	110	45	64.2±13.2	Case-control	0.08	8
Kamberi et al., (2016)	2017	IS	European	15	21	3	27	55	20	2.83–12.59	Case-control	0.40	8
Ma et al., (2017)	2017	IS	Asian	36	106	94	88	183	119	Media 66.0	Case-control	0.27	7
Lu et al., (2017)	2017	IS	Asian	31	97	89	41	109	73	NA	Case-control	0.8	7
Jiang et al., (2017)	2017	IS	Asian	36	52	18	40	54	12	57.8±10.7	Case-control	0.33	8
Kumar et al., (2016)	2016	IS	Asian	161	84	5	183	65	2	52.83±12.5	Case-control	0.14	7
Vijayan et al., (2016)	2016	IS	Asian	164	35	1	185	8	0	57.74±13.84	Case-control	0.77	8
Zhang et al., (2016)	2016	IS	Asian	13	27	10	36	18	5	NA	Case-Control	0.23	8
Herak et al., (2017)	2017	IS	European	29	34	10	41	51	8	4.3(0.01–16.7)	Case-control	0.15	7
Ranellou et al., (2015)	2015	IS	European	16	28	7	26	36	8	37.3±8.0	Case-control	0.40	8
Wei et al., (2015)	2015	IS	Asian	177	98	26	226	65	6	52.6±68.8	Case-control	0.60	8
Luo et al., (2015)	2015	IS	Asian	55	269	388	52	299	423	65.2±13.9	Case-control	0.93	8
Lv et al., (2015)	2015	IS	Asian	70	98	31	88	116	37	68.78 ± 10.63	Case-control	0.90	8
Zhou et al., (2014)	2014	IS	Asian	160	270	112	242	308	104	Media 66	Case-control	0.72	8
Atadzhanov et al., (2013)	2014	IS	African	15	8	0	96	20	0	54 ± 16	Case-control	0.31	8
Shi, (2014)	2014	IS	Asian	18	36	31	33	35	14	77.89 ± 8.85	Case-control	0.38	8
Supanc et al., (2014)	2014	IS	European	41	78	36	75	59	16	Middle-aged	Case-control	0.40	8
Fekih-Mrissa et al., (2013)	2013	IS	ONR	35	43	6	60	35	5	56.06 ± 12.5	Case-control	0.97	8
Zhang and Guo, (2012)	2012	IS	Asian	9	19	12	10	20	10	39.25 ± 4.08	Case-control	1.00	8
Djordjevic et al., (2012)	2012	IS	ONR	24	50	6	33	54	13	6.7 ± 4.9	Case-control	0.21	8
Djordjevic et al., (2012)	2012	IS	ONR	39	23	11	59	47	14	40.3 ± 11.6	Case-control	0.33	88
Somarajan et al., (2011)	2011	IS	Asian	137	65	5	129	54	5	54 ± 15.9	Case-control	0.82	8
Mohamed et al., (2011)	2011	IS	Asian	69	60	21	85	48	9	61.0 ± 10.1	Case-control	0.53	8
Hultdin et al., (2011)	2011	IS	ONR	172	114	27	401	306	59	55.0 ± 8.0	Nested	0.95	8
Salem-Berrabah et al., (2010)	2011	IS	ONR	33	15	2	57	35	5	Mean 57.62	Case-control	0.90	7
Isordia-Salas et al., (2010)	2010	IS	HLA	35	105	38	60	83	40	33.1 ± 5.8	Case-control	0.27	8
Giusti et al., (2010)	2010	IS	European	130	240	131	572	529	110	Media 44.0	Case-control	0.437	7
Tatarskyy et al., (2010)	2010	IS	ONR	73	87	23	50	45	5	64.6 ± 9.1	Case-control	0.20	8
Al-Allawi et al., (2009)	2009	IS	ONR	26	30	14	27	20	3	Media 60	Case-control	0.78	7
Sun et al., (2009)	2009	IS	Asian	45	36	38	49	37	10	>60	Case-control	0.45	7
Sabino et al., (2009)	2009	IS	HLA	12	9	0	24	12	1	33.3 ± 16.3	Case-control	0.73	7
Biswas et al., (2009)	2009	IS	Asian	24	32	2	48	10	0	<15	Case-control	0.47	7
Biswas et al., (2009))	2009	IS	Asian	67	49	4	90	30	0	Young	Case-control	0.12	8
(Morita et al., 2009)	2009	IS	European	5	6	4	48	37	5	Children	Case-control	0.53	7
Herak et al., (2009)	2009	IS	European	11	17	5	46	56	10	Children	Case-control	0.22	7
Goracy et al., (2009)	2009	IS	European	65	69	18	83	46	6	66.7 ± 12.5	Case-control	0.91	8
Zak et al., (2009)	2009	IS	European	25	30	9	32	25	2	Children	Case-control	0.27	8
Djordjevic et al., (2009)	2009	IS	ONR	9	16	1	23	22	5	Children	Case-control	0.94	8
Almawi et al., (2009)	2009	IS	ONR	54	26	38	90	26	4	NA	Case-control	0.23	7
Sawula et al., (2009)	2009	IS	European	70	50	8	35	19	5	18–101	Case-control	0.31	8
Yue et al., (2010)	2009	IS	Asian	8	19	35	14	11	5	67 ± 9	Case-control	0.29	8
Celiker et al., (2009)	2009	IS	ONR	158	4	0	63	37	6	Mean 69.8	Case-control	0.85	8
Shi et al., (2008)	2008	IS	Asian	23	45	29	20	45	34	Young	Case-control	0.07	8
Moe et al., (2008)	2008	IS	Asian	73	36	11	136	68	3	62.5 ± 1.1	Case-control	0.09	8
Gao et al., (2008)	2008	IS	Asian	4	20	18	6	11	13	≤45	Case-control	0.22	8
Zhang et al., (2008)	2008	IS	Asian	49	116	80	74	140	68	63.7 ± 10.4	Case-control	0.91	8
Berge et al., (2007)	2007	IS	European	29	125	182	25	141	163	Old	Case-control	0.47	7
Nan et al., (2007)	2007	IS	Asian	14	55	31	28	53	19	<45	case-control	0.49	8
Kim et al., (2007)	2007	IS	Asian	81	113	43	66	119	38	61.18 ± 11.09	Case-control	0.21	8
Komitopoulou et al., (2006)	2006	IS	European	36	46	8	46	39	18	5.55 ± 0.48	Case-control	0.06	8
Sazci et al., (2006)	2006	IS	ONR	42	41	9	115	119	25	53.45 ± 9.21	Case-control	0.47	8
Li et al., (2006)	2006	IS	Asian	235	184	35	190	128	16	65.20 ± 12.75	Case-control	0.34	8
Dikmen et al., (2006)	2006	IS	ONR	75	57	14	32	21	2	63.4 ± 0.87	Case-control	0.52	8
Gao et al., (2006)	2006	IS	Asian	30	49	21	32	44	24	61.08 ± 10.77	Case-control	0.25	8
Yanqun et al., (2006)	2006	IS	Asian	49	73	40	49	41	10	55 ± 6	Case-control	0.74	7
Hermans et al., (2006)	2006	IS	ONR	4	13	6	62	58	22	71.7 ± 9.3	Case-control	0.18	9
Pezzini et al., (2005)	2005	IS	European	46	83	34	60	75	23	35.0 ± 7.5	Case-control	0.96	9
En et al., (2005)	2005	IS	Asian	88	43	5	51	17	2	65 ± 10	Case-control	0.69	8
Yan et al., (2006)	2005	IS	Asian	34	24	3	37	17	3	Old	Case-control	0.58	8
Jinhuan et al., (2005)	2005	IS	Asian	55	28	4	53	24	3	Mean 65	Case-control	0.89	8
Alluri et al., (2005)	2005	IS	ONR	47	21	1	48	1	0	7–78	Case-control	0.94	8
Kawamoto et al., (2005)	2005	IS	Asian	33	43	21	91	110	40	78 ± 8.3	Case-control	0.49	8
Yi et al., (2005)	2005	IS	Asian	27	42	9	22	25	3	68.3 ± 7.6	Case-control	0.23	8
Lingling et al., (2004)	2004	IS	Asian	26	18	3	12	15	5	67.16 ± 10.11	Case-control	0.93	8
Choi et al., (2003)	2003	IS	Asian	62	97	36	73	100	25	61.4 ± 10.9	Case-control	0.30	7
Jin et al., (2004)	2003	IS	Asian	21	59	14	40	49	11	69.68 ± 8.9	Case-control	0.48	8
Jin et al., (2004)	2003	IS	Asian	15	23	21	15	11	7	NA	Case-control	0.09	7
Yan et al., (2003)	2003	IS	Asian	55	28	4	53	24	3	66.13 ± 12.54	Case-control	0.89	8
Li et al., (2002)	2002	IS	Asian	58	65	20	97	49	8	40–90	Case-control	0.58	8
Chuanqing et al., (2002)	2002	IS	Asian	24	35	10	25	35	7	62.3 ± 12.9	Case-control	0.30	8
Wenping et al., (2003)	2002	IS	Asian	8	34	19	25	46	15	61–79	Case-control	0.43	8
Huang et al., (2002)	2002	IS	Asian	11	25	13	16	24	10	55.5 ± 13.0	Case-control	0.85	8
Guangsen and Chongwen, (2002)	2002	IS	Asian	40	47	15	37	47	16	65	Case-control	0.87	8
Akar et al., (2001)	2001	IS	ONR	24	18	4	39	23	6	Children	Case-control	0.34	7
Wu et al., (2001)	2001	IS	Asian	23	40	14	92	113	24	61.4 ± 6.8	Case-control	0.21	8
Yaqin et al., (2001)	2000	IS	Asian	15	23	21	15	11	7	NA	Case-control	0.09	7
Zheng et al., (2000)	2000	IS	Asian	43	62	10	62	45	15	Mean 59	Case-control	0.14	8
Cumming et al., (1999)	1999	IS	ONR	41	6	1	42	6	0	7–36	Case-control	0.64	7
Harmon et al., (1999)	1999	IS	European	74	73	27	86	78	19	>60	Case-control	0.83	7
Gaustadnes et al., (1999)	1999	IS	European	97	88	22	545	449	90	25–68	Case-control	0.85	8
Akar et al., (1999)	1999	IS	ONR	14	10	4	63	37	6	Children	Case-control	0.85	8
Press et al., (1999)	1999	IS	ONR	72	85	10	50	57	8	65 ± 8	Case-control	0.12	8
Lalouschek et al., (1999)	1999	IS	ONR	35	37	9	32	40	9	64.76 ± 13.2	Case-control	0.50	8
Xinliang et al., (1999)	1999	IS	Asian	10	45	25	58	32	20	60.2 ± 6.1	Case-control	0.48	8
De Stefano et al., (1998)	1998	IS	European	28	27	17	65	98	35	Mean 33.9	Case-control	0.85	8
Kostulas et al., (1998)	1998	IS	ONR	50	40	10	50	40	10	NA	Case-control	0.63	7
Salooja et al., (1998)	1998	IS	European	81	76	16	114	107	21	68 (22–94)	Case-control	0.56	8
Nakata et al., (1998)	1998	IS	Asian	19	23	6	35	51	19	30–80	Case-control	0.96	8
Markus et al., (1997)	1997	IS	European	162	146	37	76	63	22	65.7 (10.5)	Case-control	0.13	7

### 3.2 Meta-analysis results

There were 34,814 participants (15,569 cases and 19,245 controls) in the 96 studies included in the meta-analysis.

#### 3.2.1 Dominant model

The TT + CT genotype showed significant heterogeneity compared with the CC genotype in the dominant genetic model (I^2^ = 69.9%; *p* < 0.001) (Table 2). The MTHFR rs1801133 (677C>T) mutation significantly increased the IS risk under the dominant genetic model (OR = 1.47; 95%CI = 1.33–1.61; *p* < 0.001). In the ancestry subgroup analysis, the rs1801133 (677C>T) polymorphism of MTHFR was evidently linked to an increased risk of IS in Asian (OR = 1.59; 95%CI = 1.41–1.80; *p* < 0.001) and Hispanic or Latin population (OR = 1.93; 95%CI = 1.39–2.67; *p* < 0.001). MTHFR rs1801133 (677C>T) gene polymorphism was associated with IS susceptibility in all age groups except young populations (middle-aged: OR = 1.59, 95%CI = 1.33–1.90, and *p* < 0.001; elderly: OR = 1.34, 95%CI = 1.17–1.53, and *p* < 0.001).

**TABLE 2:** Pooled odds ratios (ORs) and 95% confidence intervals (CIs) of the association between C677T polymorphism and stroke in the dominant model.

Model	OR (95% CI)	P_Z_	I^2^	P_H_
Dominant				
All IS	1.47 (1.33–1.61)	<0.001^a^	69.9%	<0.001
Ancestry				
Asian	1.59 (1.41–1.80)	<0.001^a^	67.2%	<0.001
European	1.35 (1.10–1.65)	0.004	69.9%	<0.001
African	2.56 (0.96–6.85)	0.061	—	—
Hispanic or Latin American	1.93 (1.39–2.67)	<0.001^a^	0.0%	0.824
Other and not reported ancestries	1.20 (0.91–1.57)	0.190	76.4%	<0.001
Age				
Young	1.46 (1.12–1.91)	0.005	52.2%	0.012
Middle-aged	1.59 (1.33–1.90)	<0.001^a^	69.8%	<0.001
Elderly	1.34 (1.17–1.53)	<0.001^a^	71.3%	<0.001
NA	1.77 (1.30–2.41)	<0.001^a^	69.3%	<0.001

Bonferroni correction for multiple testing was applied (*p*-value threshold, 0.001);

P_Z_, *p*-value for the Z-test; P_H_, *p*-value for heterogeneity;

^a^ Association was still significant after Bonferroni correction for multiple testing.

#### 3.2.2 Recessive model

The TT genotype showed significant heterogeneity compared with the CC + CT genotype in the recessive genetic model (I^2^ = 55.7%; *p* < 0.001) (Table 3). MTHFR rs1801133 (677C>T) polymorphism was associated with an increased risk of stroke under the recessive model (OR = 1.52; 95%CI = 1.36–1.71; *p* < 0.001). The ancestry subgroup analysis showed a significant difference in the Asian populations, with combined ORs of 1.58 (95% CI = 1.38–1.81; *p* < 0.001). The middle-aged and elderly people had an increased risk of stroke according to the subgroup analysis (middle-aged: OR = 1.55, 95%CI = 1.22–1.96, and *p* < 0.001; elderly: OR = 1.47, 95%CI = 1.28–1.68, and *p* < 0.001)

**TABLE 3:** Pooled odds ratios (ORs) and 95% confidence intervals (CIs) of the association between C677T polymorphism and stroke in the recessive model.

Model	Or (95% CI)	P_Z_	I^2^	P_H_
Recessive				
All IS	1.52 (1.36–1.71)	<0.001^a^	55.7%	<0.001
Ancestry				
Asian	1.58 (1.38–1.81)	<0.001^a^	47.1%	<0.001
European	1.48 (1.11–1.97)	0.007	73.1%	<0.001
African	—	—	—	—
Hispanic or Latin American	0.96 (0.68–1.37)	0.839	0.0%	0.949
Other and not reported ancestries	1.47 (1.05–2.05)	0.026	49.6%	0.005
Age				
Young	1.25 (0.81–1.92)	0.313	53.7%	0.009
Middle-aged	1.55 (1.22–1.96)	<0.001^a^	57.5%	<0.001
Elderly	1.47 (1.28–1.68)	<0.001^a^	46.8%	0.001
NA	1.99 (1.29–3.07)	<0.001^a^	64.7%	0.001

Bonferroni correction for multiple testing was applied (*p*-value threshold 0.001);

P_Z_, *p*-value for the Z-test; P_H_, *p*-value for heterogeneity;

^a^ Association was still significant after Bonferroni correction for multiple testing.

#### 3.2.3 Heterozygous model

The TC genotype showed significant heterogeneity compared with the CC genotype in the heterozygous genetic model (I^2^ = 60.4%; *p* < 0.001) (Table 4). There was an obvious association between MTHFR rs1801133 (677C>T) polymorphism and increased risk of stroke under the heterozygous model (OR = 1.36; 95%CI = 1.24–1.48; *p* < 0.001). In the subgroup analysis, MTHFR rs1801133 (677C>T) gene polymorphism was associated with stroke susceptibility in Asian (OR = 1.46; 95%CI = 1.30–1.63; *p* < 0.001), Hispanic or Latin populations (OR = 2.09; 95%CI = 1.50–2.95; *p* < 0.001), middle-aged (OR = 1.50; 95%CI = 1.27–1.78; *p* < 0.001), and elderly groups (OR = 1.23; 95%CI = 1.08–1.40; *p* = 0.001).

**TABLE 4:** Pooled odds ratios (ORs) and 95% confidence intervals (CIs) of the association between C677T polymorphism and stroke in the heterozygous model.

Model	OR (95% CI)	P_Z_	I^2^	P_H_
Heterozygote				
All IS	1.36 (1.24–1.48)	<0.001^a^	60.4%	<0.001
Ancestry				
Asian	1.46 (1.30–1.63)	<0.001^a^	58.2%	<0.001
European	1.27 (1.08–1.51)	0.005	50.4%	0.006
African	2.56 (0.96–6.85)	0.061	—	—
Hispanic or Latin American	2.09 (1.50–2.95)	<0.001^a^	0.0%	0.825
Other and not reported ancestries	1.10 (0.86–1.40)	0.453	67.6%	<0.001
Age				
Young	1.44 (1.13–1.84)	0.003	38.7%	0.069
Middle-aged	1.50 (1.27–1.78)	<0.001^a^	62.5%	<0.001
Elder	1.23 (1.08–1.40)	0.001^a^	62.8%	0.001
NA	1.48 (1.14–1.92)	0.003	50.2%	0.020

Bonferroni correction for multiple testing was applied (*p*-value threshold 0.001);

P_Z_, *p*-value for the Z-test; P_H_, *p*-value for heterogeneity;

^a^ Association was still significant after Bonferroni correction for multiple testing.

#### 3.2.4 Homozygous model

The TT genotype showed significant heterogeneity compared with the CC genotype in the homozygous genetic model (I^2^ = 64.2%; *p* < 0.001) (Table 5). There was also a significant association between MTHFR rs1801133 (677C>T) polymorphism and an increased risk of stroke under this model (OR = 1.82; 95%CI = 1.58–2.11; *p* < 0.001). However, the stratification analysis results were similar to those of the recessive model. Significant correlation was detected between MTHFR rs1801133 (677C>T) polymorphisms and the increased risk of stroke in the Asian population (OR = 1.98; 95%CI = 1.66–2.37; *p* < 0.001). Furthermore, the middle-aged and elderly people had an increased risk of stroke in the subgroup analysis (middle-aged: OR = 1.92, 95%CI = 1.45–2.53, and *p* < 0.001; elderly: OR = 1.74, 95%CI = 1.44–2.11, and *p* < 0.001).

**TABLE 5:** Pooled odds ratios (ORs) and 95% confidence intervals (CIs) of the association between C677T polymorphism and stroke in the homozygous model.

Model	Or (95% CI)	P_Z_	I^2^	P_H_
Homozygote				
All IS	1.82 (1.58–2.11)	<0.001^a^	64.2%	<0.001
Ancestry				
Asian	1.98 (1.66–2.37)	<0.001^a^	59.4%	<0.001
European	1.65 (1.14–2.39)	0.008	79.3%	<0.001
African	—	—	—	—
Hispanic or Latin American	1.60 (1.05–2.46)	0.030	0.0%	0.863
Other and not reported ancestries	1.59 (1.09–2.32)	0.017	56.2%	0.001
Age				
Young	1.43 (0.88–2.33)	0.146	55.6%	0.006
Middle-aged	1.92 (1.45–2.53)	<0.001^a^	62.7%	<0.001
Elder	1.74 (1.44–2.11)	<0.001^a^	62.3%	<0.001
NA	2.45 (1.47–4.01)	0.001^a^	69.1%	<0.001

Bonferroni correction for multiple testing was applied (*p*-value threshold 0.001);

P_Z_, *p*-value for the Z-test; P_H_, *p*-value for heterogeneity;

^a^ Association was still significant after Bonferroni correction for multiple testing.

#### 3.2.5 Allelic model

The T allele showed significant heterogeneity compared with the C allele in the allelic genetic model (I^2^ = 75.7%; *p* < 0.001) (Table 6). There was an obvious association between MTHFR rs1801133 (677C>T) polymorphism and an increased risk of stroke under the allelic model (OR = 1.37; 95%CI = 1.27–1.48; *p* < 0.001). In the subgroup analysis, MTHFR rs1801133 (677C>T) gene polymorphism was associated with stroke susceptibility in Asian populations (OR = 1.46; 95%CI = 1.33–1.60; *p* < 0.001). The middle-aged and elderly people with T allele mutation had a higher risk of stroke (middle-aged: OR = 1.42, 95%CI = 1.24–1.64, and *p* < 0.001; elderly: OR = 1.30, 95%CI = 1.18–1.43, and *p* < 0.001).

**TABLE 6:** Pooled odds ratios (ORs) and 95% confidence intervals (CIs) of the association between C677T polymorphism and stroke in the allelic model.

Model	Or (95% CI)	P_Z_	I^2^	P_H_
Allele				
All IS	1.37 (1.27–1.48)	<0.001^a^	75.7%	<0.001
Ancestry				
Asian	1.46 (1.33–1.60)	<0.001^a^	72.6%	<0.001
European	1.29 (1.09–1.53)	0.003	79.5%	<0.001
African	2.51 (1.06–5.93)	0.036	—	—
Hispanic or Latin American	1.28 (1.05–1.57)	0.016	0.0%	0.981
Other and not reported ancestries	1.26 (0.96–1.52)	0.116	81.4%	<0.001
Age				
Young	1.31 (1.05–1.64)	0.016	65.5%	<0.001
Middle-aged	1.42 (1.24–1.64)	<0.001^a^	73.7%	<0.001
Elder	1.30 (1.18–1.43)	<0.001^a^	75.0%	<0.001
NA	1.70 (1.28–2.26)	<0.001^a^	80.6%	<0.001

Bonferroni correction for multiple testing was applied (*p*-value threshold 0.001);

P_Z_, *p*-value for the Z-test; P_H_ *p*-value for heterogeneity;

^a^ Association was still significant after Bonferroni correction for multiple testing.

### 3.3 Sensitivity analysis

A sensitivity analysis was conducted to compare the pooled ORs after individually excluding each included study. There was no significant change in the results (Supplementary Figure S1).

### 3.4 Publication bias

**FIGURE 3:**
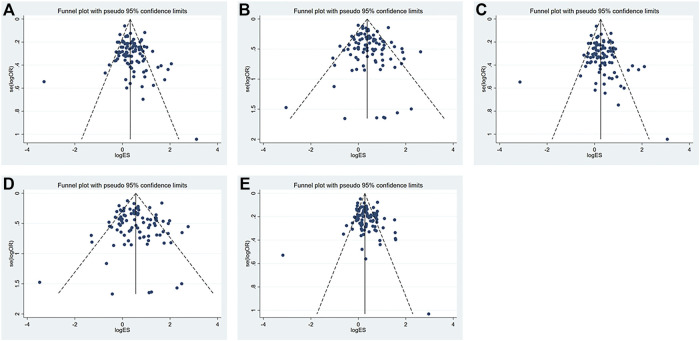
Funnel plots of the **(A)** dominant model; **(B)** recessive model; **(C)** heterozygous model; **(D)** homozygous model; and **(E)** allelic model.

The funnel plot is shown in Figure 3. All research studies included in this study distributed above the funnel plots, which indicated that variability of the effect size was low and the results were reliable. The Egger’s funnel plots for these five models were basically symmetrical though Egger’s test, indicating there was publication bias in the dominant (*p* = 0.04) and heterozygous models (*p* = 0.03) (Table 7). Nonetheless, we did not find any publication bias in all genetic models using Begg’s tests. The correlation between the lnOR and its variance and the level of heterogeneity across studies might contribute to the discrepancy between Egger’s and Begg’s tests. Actually, Begg’s test is more robust and has the appropriate type I error rates despite the sample size, the number of included studies, and the level of heterogeneity. Furthermore, when the summary estimates are ORs or RRs and there is obvious heterogeneity between studies (Schwarzer et al., 2002; Peters et al., 2006), type I error rates for Egger’s test are higher than those for Begg’s test.

**TABLE 7:** Publication bias of included studies.

IS	Egger’s test			Begg’s test	
	Co-efficiency	Standard error	*p*-value	Z	*p*-value
Dominant model	0.83	0.39	0.04^a^	1.56	0.12
Recessive model	0.37	0.29	0.20	1.28	0.20
Heterozygote model	0.74	0.34	0.03^a^	1.83	0.07
Homozygote model	0.28	0.36	0.43	1.18	0.24
Allele model	0.76	0.44	0.09	1.71	0.09

^a^ *p* < 0.05.

## 4 Discussion

This meta-analysis demonstrates associations between MTHFR rs1801133 (677C>T) genetic polymorphism and susceptibility to IS under all genetic models. Our results were consistent with a previous meta-analysis performed in 2016, wherein this polymorphism was found to be potentially involved in the development of IS (Song et al., 2016). This suggests the MTHFR C677T mutation is a genetic risk factor of IS, and primary and secondary prevention should be initiated in a timely manner in population with this mutation.

Several factors might explain the association of the MTHFR rs1801133 (677C>T) mutation and increased IS risk. Most importantly, the MTHFR rs1801133 (677C>T) mutation leads to decreased MTHFR activity and elevated homocysteine levels (Castro et al., 2004). Hyperhomocysteinemia is linked to the overproduction of free radicals (Xi et al., 2016), induction of oxidative stress (Esse et al., 2019; Tchantchou et al., 2021), endothelial injury (Salvio et al., 2021), coagulation, and lipid metabolism disturbance (Herrmann, 2001), which all contribute to the incidence of IS. Meanwhile, previous studies demonstrated that people with the MTHFR rs1801133 (677C>T) mutation show a poor response to homocysteine-lowering treatment (Qin et al., 2020). Furthermore, a meta-analysis published in the current year showed that MTHFR rs1801133 (677C>T) polymorphism is related to susceptibility to H-type hypertension (Liao et al., 2022), which is a traditional risk factor for IS.

We also conducted a subgroup analysis on the basis of age and ancestry of the study population to further understand the significance of the MTHFR rs1801133 (677C>T) mutation in various populations. The result of subgroup analysis showed increased IS risk in populations with the MTHFR rs1801133 (677C>T) mutation in middle-aged and elderly groups. It was consistent with the fact that older people were more susceptible to atherosclerosis, which was an important cause of IS. Previous studies had demonstrated that the MTHFR rs1801133 (677C>T) mutation increased the risk of IS in patients with large-artery atherosclerosis (Cui, 2016) and adults (Xin et al., 2009).

Another important finding of this meta-analysis was the stable association between IS risk and MTHFR rs1801133 (677C>T) polymorphism in Asian populations in all genetic models and Hispanic or Latin American population in dominant and heterozygous models. Similar trends were also found among other populations, although no statistically significant difference was found in some genetic models. This may be related to the following reasons: 1) the frequency of the MTHFR 677T gene variant differs among ethnic groups due to different genetic backgrounds. Previous studies indicated that the frequency of the MTHFR rs1801133 T allele was 24–40% in Europeans, 40% in Koreans, and 26–37% in Japanese (Shao et al., 2017); 2) MTHFR rs1801133 (677C>T) was associated with increased coronary heart disease only when the folate level was low (Klerk et al., 2002). Thus, various dietary habits and differences in folate intake may also contribute to this difference; 3) the difference in the power of included studies may be another cause of this result. Nonetheless, the results for these ancestry groups need to be interpreted with caution, and more high-quality studies are still required to explore the correlation between MTHFR rs1801133 (677C>T) polymorphism and IS risk in these ancestry groups.

This study has several strengths. First, we included the most recent and relevant studies in this meta-analysis. In addition, we further analyzed the association between MTHFR rs1801133 (677C>T) polymorphism and IS risk in different populations. Finally, this meta-analysis included high-quality observational studies using real-world data with a large number of patients.

However, this meta-analysis also has some limitations. First, the study was based on the secondary study-level data. Age groups were defined according to the mean or median age of study subjects, and some studies did not provide clear information on age. Thus, the age stratification of subgroups might not be accurate. Second, the findings of this meta-analysis were mainly based on case-control studies and lacked prospective research; therefore, they should be interpreted with caution.

## 5 Conclusion

Our findings showed that the MTHFR rs1801133 (677C>T) variant may contribute to an increased risk of IS. This association was statistically significant in the Asian and Hispanic or Latin American cohorts and showed a similar trend in the populations of other ancestries. For middle-aged and elderly people, MTHFR rs1801133 (677C>T) might be a promising biomarker for early detection and prediction of the prognosis of IS. However, high-quality, prospective studies are needed in the future.

## References

[B1] Akar N. Akar E. Deda G. Sipahi T. OrsAl A. (1999). Factor V1691 G-A, prothrombin 20210 G-A, and methylenetetrahydrofolate reductase 677 C-T variants in Turkish children with cerebral infarct. J. Child. Neurol. 14 (11), 749–751. 10.1177/088307389901401113 10593555

[B2] Akar N. Akar E. Ozel D. Deda G. Sipahi T. (2001). Common mutations at the homocysteine metabolism pathway and pediatric stroke. Thromb. Res. 102 (2), 115–120. 10.1016/s0049-3848(01)00226-2 11323021

[B3] Al-Allawi N. S. Avo A. S. Jubrael J. S. (2009). Methylenetetrahydrofolate reductase C677T polymorphism in Iraqi patients with ischemic stroke. Neurol. India 57 (5), 631–634. 19934565 10.4103/0028-3886.57821

[B4] Alluri R. V. Mohan V. Komandur S. Chawda K. Chaudhuri J. R. Hasan Q. (2005). MTHFR C677T gene mutation as a risk factor for arterial stroke: A hospital based study. Eur. J. Neurol. 12 (1), 40–44. 10.1111/j.1468-1331.2004.00938.x 15613145

[B5] Almawi W. Y. Khan A. Al-Othman S. S. Bakhiet M. (2009). Case-control Study of methylenetetrahydrofolate reductase mutations and hyperhomocysteinemia and risk of stroke. J. Stroke Cerebrovasc. Dis. 18 (5), 407–408. 10.1016/j.jstrokecerebrovasdis.2008.12.003 19717029

[B6] Atadzhanov M. Mwaba M. H. Mukomena P. N. Lakhi S. Rayaprolu S. Ross O. A. (2013). Association of the APOE, MTHFR and ACE genes polymorphisms and stroke in Zambian patients. Neurol. Int. 5 (4), e20–e72. 10.4081/ni.2013.e20 24416484 PMC3883065

[B7] Berge E. Haug K. B. F. Sandset E. C. Haugbro K. K. Turkovic M. Sandset P. M. (2007). The factor V Leiden, prothrombin gene 20210GA, methylenetetrahydrofolate reductase 677CT and platelet glycoprotein IIIa 1565TC mutations in patients with acute ischemic stroke and atrial fibrillation. Stroke 38 (3), 1069–1071. 10.1161/01.STR.0000258076.04860.8e 17290027

[B8] Biswas A. Ranjan R. Meena A. Akhter M. S. Yadav B. K. Munisamy M. (2009). Homocystine levels, polymorphisms and the risk of ischemic stroke in young Asian Indians. J. Stroke Cerebrovasc. Dis. 18 (2), 103–110. 10.1016/j.jstrokecerebrovasdis.2008.09.014 19251185

[B9] Black M. Wang W. Wang W. (2015). Ischemic stroke: From next generation sequencing and GWAS to community genomics? Omics 19 (8), 451–460. 10.1089/omi.2015.0083 26230531

[B10] Brattström L. Wilcken D. E. Ohrvik J. Brudin L. (1998). Common methylenetetrahydrofolate reductase gene mutation leads to hyperhomocysteinemia but not to vascular disease: The result of a meta-analysis. Circulation 98 (23), 2520–2526. 10.1161/01.cir.98.23.2520 9843457

[B11] Castro R. Rivera I. Ravasco P. Camilo M. E. Jakobs C. Blom H. J. (2004). 5, 10-methylenetetrahydrofolate reductase (MTHFR) 677C-->T and 1298A-->C mutations are associated with DNA hypomethylation. J. Med. Genet. 41 (6), 454–458. 10.1136/jmg.2003.017244 15173232 PMC1735802

[B12] Celiker G. Can U. Verdi H. Yazici A. C. Ozbek N. Atac F. B. (2009). Prevalence of thrombophilic mutations and ACE I/D polymorphism in Turkish ischemic stroke patients. Clin. Appl. Thromb. Hemost. 15 (4), 415–420. 10.1177/1076029608315163 18387982

[B13] Cernera G. Comegna M. Gelzo M. Savoia M. Bruzzese D. Mormile M. (2021). Molecular analysis of prothrombotic gene variants in patients with acute ischemic stroke and with transient ischemic attack. Med. Kaunas. Lith. 57, 723. 10.3390/medicina57070723 PMC8306646 34357004

[B14] Chang G. Kuai Z. Wang J. Wu J. Xu K. Yuan Y. (2019). The association of MTHFR C677T variant with increased risk of ischemic stroke in the elderly population: A meta-analysis of observational studies. BMC Geriatr. 19 (1), 331. 10.1186/s12877-019-1304-y 31775641 PMC6882223

[B15] Choi B. O. Kim N. K. Kim S. H. Kang M. S. Lee S. Ahn J. Y. (2003). Homozygous C677T mutation in the MTHFR gene as an independent risk factor for multiple small-artery occlusions. Thromb. Res. 111 (1-2), 39–44. 10.1016/j.thromres.2003.08.022 14644077

[B16] Chuanqing T. Minaqing H. Jianzeng W. Sizhong X. Chunying X. Chuyan P. (2002). The relation of methylenetetradrofolate reductase gene Mutation and Cystathionine β-synthase Gene Mutation Exist in cerebral infarction. Thrombosis hemostasis 04, 149–151.

[B17] Cui T. (2016). MTHFR C677T mutation increased the risk of ischemic stroke, especially in large-artery atherosclerosis in adults: An updated meta-analysis from 38 researches. Int. J. Neurosci. 126 (1), 10–19. 10.3109/00207454.2014.990559 25453894

[B18] Cumming A. M. Olujohungbe A. Keeney S. SingH H. Hay C. R. Serjeant G. R. (1999). The methylenetetrahydrofolate reductase gene C677T polymorphism in patients with homozygous sickle cell disease and stroke. Br. J. Haematol. 107 (3), 569–571. 10.1046/j.1365-2141.1999.01728.x 10583261

[B19] De Stefano V. Chiusolo P. Paciaroni K. CasorellI I. Rossi E. MolinariM. (1998). Prothrombin G20210A mutant genotype is a risk factor for cerebrovascular ischemic disease in young patients. Blood 91 (10), 3562–3565. 10.1182/blood.v91.10.3562.3562_3562_3565 9572989

[B20] Dikmen M. Ozbabalik D. Gunes H. V. DegIrmencI I. Bal C. Ozdemir G. (2006). Acute stroke in relation to homocysteine and methylenetetrahydrofolate reductase gene polymorphisms. Acta Neurol. Scand. 113 (5), 307–314. 10.1111/j.1600-0404.2005.00556.x 16629766

[B21] Djordjevic V. Stankovic M. Brankovic-Sreckovic V. Rakicevic L. Damnjanovic T. Antonijevic N. (2012). Prothrombotic genetic risk factors in stroke: A possible different role in pediatric and adult patients. Clin. Appl. Thromb. Hemost. 18 (6), 658–661. 10.1177/1076029611432136 22275392

[B22] Djordjevic V. Stankovic M. Brankovic-Sreckovic V. Rakicevic L. Radojkovic D. (2009). Genetic risk factors for arterial ischemic stroke in children: A possible MTHFR and eNOS gene-gene interplay? J. Child. Neurol. 24 (7), 823–827. 10.1177/0883073808330164 19372095

[B23] En X. Bingmei D. Shengqiang C. Haifeng X. Xuefen L. (2005). The relationship between Gene Polymorphisms of MTRR A66G, MS D919G, MTHFR C677T and Cerebral Infarction. Chin. J. Neuromed 09, 902–904+907.

[B24] Esse R. Barroso M. Tavares de Almeida I. Castro R. (2019). The contribution of homocysteine metabolism disruption to endothelial dysfunction: State-of-the-Art. Int. J. Mol. Sci. 20, 867. 10.3390/ijms20040867 30781581 PMC6412520

[B25] Fekih-Mrissa N. (2013). Role of methylenetetrahydrofolate reductase A1298C polymorphism in cerebral venous thrombosis. Blood Coagulation Fibrinolysis 24 (2), 118–119. 23314385 10.1097/MBC.0b013e32835707cd

[B26] Francis J. Raghunathan S. Khanna P. (2007). The role of genetics in stroke. Postgrad. Med. J. 83 (983), 590–595. 10.1136/pgmj.2007.060319 17823225 PMC2600010

[B27] Gao J. Sun Y. Li Y. Su L. Guo L. (2008). Hyperhomocysteine genetic polymorphism of methylenetetrahydrofolate reductase in young adults with ischemic stroke. J. Zhengzhou Uiversity Med. Sci. 03, 570–573. 10.13705/j.issn.1671-6825.2008.03.052

[B28] Gao X. Yang H. ZhiPing T. (2006). Association studies of genetic polymorphism, environmental factors and their interaction in ischemic stroke. Neurosci. Lett. 398 (3), 172–177. 10.1016/j.neulet.2005.12.078 16443328

[B29] Gaustadnes M. RudigerN. Moller J. Rasmussen K. Bjerregaard Larsen T. Ingerslev J. (1999). Thrombophilic predisposition in stroke and venous thromboembolism in Danish patients. Blood Coagul. Fibrinolysis 10 (5), 251–259. 10.1097/00001721-199907000-00006 10456616

[B30] Giusti B. Saracini C. Bolli P. Magi A. Martinelli I. Peyvandi F. (2010). Early-onset ischaemic stroke: Analysis of 58 polymorphisms in 17 genes involved in methionine metabolism. Thromb. Haemost. 104 (2), 231–242. 10.1160/TH09-11-0748 20458436

[B31] Goracy I. Cyrylowski L. KaczMarczykM. FabiAn A. Koziarska D. Goracy J. (2009). C677T polymorphism of the methylenetetrahydrofolate reductase gene and the risk of ischemic stroke in Polish subjects. J. Appl. Genet. 50 (1), 63–67. 10.1007/BF03195654 19193985

[B32] Goyette P. Sumner J. S. Milos R. Duncan A. M. Rosenblatt D. S. Matthews R. G. (1994). Human methylenetetrahydrofolate reductase: Isolation of cDNA, mapping and mutation identification. Nat. Genet. 7 (2), 195–200. 10.1038/ng0694-195 7920641

[B33] Guangsen Z. Chongwen D. (2002). Correlation analysis between plasma homocysteine level and polymorphism of homocysteine metabolism re- lated enzymes in ischemic cerebrovascular or cardiovascular diseases. Chin. J. Hematol., 13–16. 12015064

[B34] Harmon D. L. Doyle R. M. Meleady R. DoyleM. Shields D. C. BaRRy R. (1999). Genetic analysis of the thermolabile variant of 5, 10-methylenetetrahydrofolate reductase as a risk factor for ischemic stroke. Arterioscler. Thromb. Vasc. Biol. 19 (2), 208–211. 10.1161/01.atv.19.2.208 9974399

[B35] Herak D. C. Antolic M. R. Krleza J. L. Pavic M. Dodig S. Duranovic V. (2009). Inherited prothrombotic risk factors in children with stroke, transient ischemic attack, or migraine. Pediatrics 123, e653–e660. 10.1542/peds.2007-3737 19336355

[B36] Herak D. C. Lenicek Krleza J. Radic Antolic M. Horvat I. Djuranovic V. Zrinski Topic R. (2017). Association of polymorphisms in coagulation factor genes and enzymes of homocysteine metabolism with arterial ischemic stroke in children. Clin. Appl. Thromb. Hemost. 23 (8), 1042–1051. 10.1177/1076029616672584 28301901

[B37] Hermans M. P. Gala J. L. Buysschaert M. (2006). The MTHFR CT polymorphism confers a high risk for stroke in both homozygous and heterozygous T allele carriers with Type 2 diabetes. Diabet. Med. 23 (5), 529–536. 10.1111/j.1464-5491.2006.01841.x 16681562

[B38] Herrmann W. (2001). The importance of hyperhomocysteinemia as a risk factor for diseases: An overview. Clin. Chem. Lab. Med. 39 (8), 666–674. 10.1515/CCLM.2001.110 11592431

[B39] Hou J. Zeng X. Xie Y. Wu H. Zhao P. (2018). Genetic polymorphisms of methylenetetrahydrofolate reductase C677T and risk of ischemic stroke in a southern Chinese Hakka population. Med. (United States) 97, e13645. 10.1097/MD.0000000000013645 PMC6320192 30572478

[B40] Huang L. W. Li L. L. Li J. Chen X. R. Yu M. (2022). Association of the methylenetetrahydrofolate reductase (MTHFR) gene variant C677T with serum homocysteine levels and the severity of ischaemic stroke: A case-control study in the southwest of China. J. Int. Med. Res. 50 (2), 3000605221081632. 10.1177/03000605221081632 35225709 PMC8894968

[B41] Huang Y. Zhao Yl Y. Li S. (2002). Hyperhomocysteine, methylenetetrahydrofolate reductase gene, and other risk factors in ischemic stroke. Zhonghua Yi Xue Za Zhi 82 (2), 119–122. 11953142

[B42] Hultdin J. Van Guelpen B. Winkvist A. Hallmans G. Weinehall L. Stegmayr B. (2011). Prospective study of first stroke in relation to plasma homocysteine and MTHFR 677C>T and 1298A>C genotypes and haplotypes - evidence for an association with hemorrhagic stroke. Clin. Chem. Lab. Med. 49 (9), 1555–1562. 10.1515/CCLM.2011.234 21631392

[B43] Isordia-Salas I. Barinagarrementería-Aldatz F. Leaños-Miranda A. Borrayo-Sánchez G. Vela-Ojeda J. García-Chávez J. (2010). The C677T polymorphism of the methylenetetrahydrofolate reductase gene is associated with idiopathic ischemic stroke in the young Mexican-Mestizo population. Cerebrovasc. Dis. 29 (5), 454–459. 10.1159/000289349 20203488

[B44] Jiang S. Li J. Zhang Y. Venners S. A. Tang G. Wang Y. (2017). Methylenetetrahydrofolate reductase C677T polymorphism, hypertension and risk of stroke: A prospective, nested case-control study. Int. J. Neurosci. 127 (3), 253–260. 10.1080/00207454.2016.1183126 27126505

[B45] Jiménez-González M. C. Santiago-German D. Castillo-Henkel E. F. Alvarado-Moreno J. A. Hernandez-Juarez J. Leanos-Miranda A. (2021). Identification of genetic risk factors associated with ischaemic stroke in young Mexican patients. Neurologia 36 (5), 337–345. 10.1016/j.nrleng.2018.01.011 34714231

[B46] Jin Z. Lei L. Hong S. Qingqing J. Qing W. Weiping J. (2004). The relationship between MTHFR gene polymorphism and cerebral hemorrhage. J. Clin. Neurol. 4, 267–269.

[B63] Jin Z. Lei L. Hong S. Qingqing J. Qing W. Weiping J. (2004). Relation between MTHFR and brain infarction. Med. J. CASC, 267–269. 10.13705/j.issn.1671-6825.2008.03.052

[B47] Jinhuan C. Guang Y. Yan S. (2005). The study of MTHFR gene C677T polymorphism in the case of cerebral thrombosis. JP Mt., 968–970.

[B48] Kamberi B. Kamberi F. Spiroski M. (2016). Vascular genetic variants and ischemic stroke susceptibility in albanians from the Republic of Macedonia. Open Access Maced. J. Med. Sci. 4 (4), 556–564. 10.3889/oamjms.2016.114 28028391 PMC5175499

[B49] Kawamoto R. Kohara K. Oka Y. Tomita H. Tabara Y. Miki T. (2005). An association of 5, 10-methylenetetrahydrofolate reductase (MTHFR) gene polymorphism and ischemic stroke. J. Stroke Cerebrovasc. Dis. 14 (2), 67–74. 10.1016/j.jstrokecerebrovasdis.2004.12.003 17904003

[B50] Kim O. J. Hong S. P. Ahn J. Y. Hong S. H. Hwang T. S. Kim S. O. (2007). Influence of combined methionine synthase (MTR 2756A > G) and methylenetetrahydrofolate reductase (MTHFR 677C > T) polymorphisms to plasma homocysteine levels in Korean patients with ischemic stroke. Yonsei Med. J. 48 (2), 201–209. 10.3349/ymj.2007.48.2.201 17461517 PMC2628129

[B51] Klerk M. Verhoef P. Clarke R. Blom H. J. Kok F. J. Schouten E. G. (2002). MTHFR 677C-->T polymorphism and risk of coronary heart disease: A meta-analysis. JAMA 288 (16), 2023–2031. 10.1001/jama.288.16.2023 12387655

[B52] Komitopoulou A. Kapsimali Z. Pergantou H. Adamtziki E. AroniS S. (2006). Mutations and polymorphisms in genes affecting hemostasis proteins and homocysteine metabolism in children with arterial ischemic stroke. Cerebrovasc. Dis. 22 (1), 13–20. 10.1159/000092332 16567932

[B53] Kostulas K. CrisbyM. Huang W. X. LannfeLt L. HagenfeLdt L. Eggertsen G. (1998). A methylenetetrahydrofolate reductase gene polymorphism in ischaemic stroke and in carotid artery stenosis. Eur. J. Clin. Invest. 28 (4), 285–289. 10.1046/j.1365-2362.1998.00281.x 9615905

[B54] Kumar A. Misra S. Hazarika A. Kumar P. Sagar R. Pathak A. (2016). Association between methylenetetrahydrofolate reductase (MTHFR) C677T gene polymorphism and risk of ischemic stroke in north Indian population: A hospital based case–control study. Egypt. J. Med. Hum. Genet. 17 (4), 359–365. 10.1016/j.ejmhg.2016.01.001

[B55] Lalouschek W. Aull S. Serles W. Schnider P. Mannhalter C. PabInger-FaschIng I. (1999). C677T MTHFR mutation and factor V leiden mutation in patients with TIA/minor stroke: A case-control study. Thromb. Res. 93 (2), 61–69. 10.1016/s0049-3848(98)00154-6 9950259

[B56] Li A. Shi Y. Xu L. Zhang Y. Zhao H. Li Q. (2017). A possible synergistic effect of MTHFR C677T polymorphism on homocysteine level variations increased risk for ischemic stroke. Med. (United States) 96, e9300. 10.1097/MD.0000000000009300 PMC5758196 29390494

[B57] Li C. M. Zhang C. Lu X. l. Feng H. y. Su Q. x. Zeng Y. (2006). Relationship between methylenetrahydrofolate reductase gene and ischemic stroke. Chin. Crit. Care Med. 18 (5), 264–267. 16700986

[B58] Li C. Zhang C. Qiu S. Lu X. Zeng Y. Wu H. (2002). Polymorphisms of ACE-1 and MTHFR genes and genetic susceptibility of ischemic stroke. Zhonghua Yi Xue Za Zhi 82 (15), 1046–1049. 12194796

[B59] Liao S. Guo S. Ma R. He J. Yan Y. Zhang X. (2022). Association between methylenetetrahydrofolate reductase (MTHFR) C677T polymorphism and H-type hypertension: A systematic review and meta-analysis. Ann. Hum. Genet. 86, 278–289. 10.1111/ahg.12468 35394066

[B60] Liew S. C. Gupta E. D. (2015). Methylenetetrahydrofolate reductase (MTHFR) C677T polymorphism: Epidemiology, metabolism and the associated diseases. Eur. J. Med. Genet. 58 (1), 1–10. 10.1016/j.ejmg.2014.10.004 25449138

[B61] Lingling H. (2004). Correlation between C677T mutation of MT HF R gene and cerebral infarction. Jiangsu Med. J., 861–862. 10.19460/j.cnki.0253-3685.2004.11.031

[B62] Linnebank M. Moskau S. Semmler A. Hoefgen B. Bopp G. Kallweit U. (2012). A possible genetic link between MTHFR genotype and smoking behavior. PLoS One 7, e53322. 10.1371/journal.pone.0053322 23285280 PMC3532068

[B64] Lu Z. Xiaoyan C. Boai Z. (2017). Analysis of relationship of plasma homocysteine level and polymorphism of methylenetetrahydrofolate reductase with ischemic stroke. Chin. J. Stroke 12 (05), 404–409.

[B65] Luo M. Li J. Sun X. Lai R. Wang Y. Xu X. (2015). Interactions among candidate genes selected by meta-analyses resulting in higher risk of ischemic stroke in a Chinese population. PLoS ONE 10, e0145399. 10.1371/journal.pone.0145399 26710338 PMC4692506

[B66] Lv Q. Q. Lu J. Sun H. Zhang J. S. (2015). Association of methylenetetrahydrofolate reductase (MTHFR) gene polymorphism with ischemic stroke in the Eastern Chinese Han population. Genet. Mol. Res. 14 (2), 4161–4168. 10.4238/2015.April.27.31 25966188

[B67] Ma L. Jiang Y. Kong X. Yan M. Zhao T. Zhao H. (2017). Synergistic effect of the MTHFR C677T and EPHX2 G860A polymorphism on the increased risk of ischemic stroke in Chinese type 2 diabetic patients. J. Diabetes Res. 2017, 6216205. 10.1155/2017/6216205 28409162 PMC5376931

[B68] Malik R. Chauhan G. Traylor M. Sargurupremraj M. Okada Y. Mishra A. (2019). Publisher Correction: Multiancestry genome-wide association study of 520, 000 subjects identifies 32 loci associated with stroke and stroke subtypes. Nat. Genet. 51 (7), 1192–1193. 10.1038/s41588-019-0449-0 31160810

[B69] Malik R. Dichgans M. (2018). Challenges and opportunities in stroke genetics. Cardiovasc. Res. 114 (9), 1226–1240. 10.1093/cvr/cvy068 29554300

[B70] Mao X. Han L. (2018). The relationship of methylenetetrahydrofolate reductase gene C677T polymorphism and ischemic stroke in Chinese han population. Ann. Clin. Lab. Sci. 48 (2), 242–247. 29678854

[B71] Markus H. S. AliN. Swaminathan R. SankArAlingAm A. Molloy J. Powell J. (1997). A common polymorphism in the methylenetetrahydrofolate reductase gene, homocysteine, and ischemic cerebrovascular disease. Stroke 28 (9), 1739–1743. 10.1161/01.str.28.9.1739 9303018

[B72] Mazdeh M. Khazaie M. Omrani M. D. Noroozi R. Komaki A. Karimi M. (2021). Association between methylene tetrahydrofolate reductase polymorphisms and risk of ischemic stroke. Int. J. Neurosci. 131 (1), 44–48. 10.1080/00207454.2020.1733554 32098547

[B73] Moe K. T. Woon F. P. De Silva D. A. Wong P. Koh T. H. Kingwell B. (2008). Association of acute ischemic stroke with the MTHFR C677T polymorphism but not with NOS3 gene polymorphisms in a Singapore population. Eur. J. Neurol. 15 (12), 1309–1314. 10.1111/j.1468-1331.2008.02308.x 19049547

[B74] Mohamed E. H. M. Tan K. S. Ali J. M. Mohamed Z. (2011). TT genotype of the methylenetetrahydrofolate reductase C677T polymorphism is an important determinant for homocysteine levels in multi-ethnic Malaysian ischaemic stroke patients. Ann. Acad. Med. Singap. 40 (4), 186–191. 21678004

[B75] Moher D. Liberati A. Tetzlaff J. Altman D. G. (2009). Preferred reporting items for systematic reviews and meta-analyses: The PRISMA statement. Bmj 339, b2535. 10.1136/bmj.b2535 19622551 PMC2714657

[B76] Morita D. C. Donaldson A. Butterfield R. J. Benedict S. L. Bale J. F. Jr (2009). Methylenetetrahydrofolate reductase gene polymorphism and childhood stroke. Pediatr. Neurol. 41 (4), 247–249. 10.1016/j.pediatrneurol.2009.04.017 19748043

[B77] Nakata Y. KaTsuya T. Takami S. SatoN. Fu Y. IshiKawa K. (1998). Methylenetetrahydrofolate reductase gene polymorphism: Relation to blood pressure and cerebrovascular disease. Am. J. Hypertens. 11 (8), 1019–1023. 10.1016/s0895-7061(98)00046-6 9715796

[B78] Nan G. Wang L. Bu S. (2007). The association of MTHFR A1298C and CBS G919A gene mutations and ischemic stroke in young adults. Chin. J. Lab. Diagn, 1697–1699.

[B79] Peters J. L. Sutton A. J. Jones D. R. Abrams K. R. Rushton L. (2006). Comparison of two methods to detect publication bias in meta-analysis. Jama 295 (6), 676–680. 10.1001/jama.295.6.676 16467236

[B80] Pezzini A. Grassi M. Del Zotto E. Archetti S. Spezi R. Vergani V. (2005). Cumulative effect of predisposing genotypes and their interaction with modifiable factors on the risk of ischemic stroke in young adults. Stroke 36 (3), 533–539. 10.1161/01.STR.0000155741.31499.c2 15692115

[B81] Phipps M. S. Cronin C. A. (2020). Management of acute ischemic stroke. Bmj 368, l6983. 10.1136/bmj.l6983 32054610

[B82] Prabhakaran S. Ruff I. Bernstein R. A. (2015). Acute stroke intervention: A systematic review. JAMA 313 (14), 1451–1462. 10.1001/jama.2015.3058 25871671

[B83] Press R. D. BeamerN. EvAns A. DeLoughery T. G. Coull B. M. (1999). Role of a common mutation in the homocysteine regulatory enzyme methylenetetrahydrofolate reductase in ischemic stroke. Diagn. Mol. Pathol. 8 (1), 54–58. 10.1097/00019606-199903000-00009 10408794

[B84] Qin X. Spence J. D. Li J. Zhang Y. Li Y. Sun N. (2020). Interaction of serum vitamin B(12) and folate with MTHFR genotypes on risk of ischemic stroke. Neurology 94, e1126–e1136. 10.1212/WNL.0000000000008932 31932513 PMC7220236

[B85] Ranellou K. Paraskeva A. Kyriazopoulos P. Batistatou A. Evangelou A. El-Aly M. (2015). Polymorphisms in prothrombotic genes in young stroke patients in Greece: A case-controlled study. Blood Coagul. Fibrinolysis 26 (4), 430–435. 10.1097/MBC.0000000000000274 25699610

[B86] Rozen R. (1997). Genetic predisposition to hyperhomocysteinemia: Deficiency of methylenetetrahydrofolate reductase (MTHFR). Thromb. Haemost. 78 (1), 523–526. 10.1055/s-0038-1657581 9198208

[B87] Sabino A. Fernandes A. P. Lima L. M. Ribeiro D. D. Sousa M. O. de Castro Santos M. E. R. (2009). Polymorphism in the methylenetetrahydrofolate reductase (C677T) gene and homocysteine levels: A comparison in Brazilian patients with coronary arterial disease, ischemic stroke and peripheral arterial obstructive disease. J. Thromb. Thrombolysis 27 (1), 82–87. 10.1007/s11239-007-0172-z 18040753

[B88] Salem-Berrabah O. B. Mrissa R. Machghoul S. Hamida A. B. N'siri B. Mazigh C. (2010). Hyperhomocysteinemia, C677T MTHFR polymorphism and ischemic stroke in Tunisian patients. Tunis. Med. 88 (9), 655–659. 20812180

[B89] Salomi B. S. B. Solomon R. Turaka V. P. Aaron S. Christudass C. S. (2021). Cryptogenic stroke in the young: Role of candidate gene polymorphisms in Indian patients with ischemic etiology. Neurol. India 69 (6), 1655–1662. 10.4103/0028-3886.333441 34979665

[B90] Salooja N. Catto A. Carter A. Tudenham E. G. Grant P. J. (1998). Methylene tetrahydrofolate reductase C677T genotype and stroke. Clin. Lab. Haematol. 20 (6), 357–361. 10.1046/j.1365-2257.1998.00158.x 9951581

[B91] Salvio G. Ciarloni A. Cutini M. Balercia G. (2021). Hyperhomocysteinemia: Focus on endothelial damage as a cause of erectile dysfunction. Int. J. Mol. Sci. 22, 418. 10.3390/ijms22010418 33401548 PMC7795368

[B92] Sawula W. Banecka-Majkutewicz Z. Kadzinski L. Jakobkiewicz-Banecka J. Wegrzyn G. Nyka W. (2009). Homocysteine level and metabolism in ischemic stroke in the population of Northern Poland. Clin. Biochem. 42 (6), 442–447. 10.1016/j.clinbiochem.2008.12.019 19166826

[B93] Sazci A. Ergul E. Tuncer N. Akpinar G. Kara I. (2006). Methylenetetrahydrofolate reductase gene polymorphisms are associated with ischemic and hemorrhagic stroke: Dual effect of MTHFR polymorphisms C677T and A1298C. Brain Res. Bull. 71 (1-3), 45–50. 10.1016/j.brainresbull.2006.07.014 17113927

[B94] Schwarzer G. Antes G. Schumacher M. (2002). Inflation of type I error rate in two statistical tests for the detection of publication bias in meta-analyses with binary outcomes. Stat. Med. 21 (17), 2465–2477. 10.1002/sim.1224 12205693

[B95] Shao W. Yuan Y. Li Y. (2017). Association between MTHFR C677T polymorphism and methotrexate treatment outcome in rheumatoid arthritis patients: A systematic review and meta-analysis. Genet. Test. Mol. Biomarkers 21 (5), 275–285. 10.1089/gtmb.2016.0326 28277784

[B96] Sharp L. Little J. (2004). Polymorphisms in genes involved in folate metabolism and colorectal neoplasia: A HuGE review. Am. J. Epidemiol. 159 (5), 423–443. 10.1093/aje/kwh066 14977639

[B97] Shi C. Kang X. Wang Y. Zhou Y. (2008). The coagulation factor V Leiden, MTHFRC677T variant and eNOS 4ab polymorphism in young Chinese population with ischemic stroke. Clin. Chim. Acta. 396 (1-2), 7–9. 10.1016/j.cca.2008.06.009 18602910

[B98] Shi M. (2014). The correlation of homocysteine concentration and MTHFR gene polymorphism with ischemic stroke. China: Zhenzhou University.

[B99] Somarajan B. I. Kalita J. Mittal B. Misra U. K. (2011). Evaluation of MTHFR C677T polymorphism in ischemic and hemorrhagic stroke patients. A case-control study in a Northern Indian population. J. Neurol. Sci. 304 (1-2), 67–70. 10.1016/j.jns.2011.02.010 21406306

[B100] Song Y. Li B. Wang C. Wang P. Gao X. Liu G. (2016). Association between 5, 10-methylenetetrahydrofolate reductase C677T gene polymorphism and risk of ischemic stroke: A meta-analysis. J. Stroke Cerebrovasc. Dis. 25 (3), 679–687. 10.1016/j.jstrokecerebrovasdis.2015.11.041 26776436

[B101] Stang A. (2010). Critical evaluation of the Newcastle-Ottawa scale for the assessment of the quality of nonrandomized studies in meta-analyses. Eur. J. Epidemiol. 25 (9), 603–605. 10.1007/s10654-010-9491-z 20652370

[B102] Sun J. Z. Xu Y. Lu H. Zhu Y. (2009). Polymorphism of the methylenetetrahydrofolate reductase gene association with homocysteine and ischemic stroke in type 2 diabetes. Neurol. India 57 (5), 589–593. 10.4103/0028-3886.57808 19934557

[B103] Supanc V. Sonicki Z. Vukasovic I. Solter V. V. Zavoreo I. Kes V. B. (2014). The role of classic risk factors and prothrombotic factor gene mutations in ischemic stroke risk development in young and middle-aged individuals. J. Stroke Cerebrovasc. Dis. 23, e171–e176. 10.1016/j.jstrokecerebrovasdis.2013.09.025 24189452

[B104] Tatarskyy P. F. Kucherenko A. M. Kravchenko S. A. Shulzenko D. V. Kuznetsova S. M. Livshits L. A. (2010). Ischemic stroke in Ukrainian population: Possible involvement of the F2 G20210A, F5 G1691A and MTHFR C677T gene variants. Biopolym. Cell 26, 299–305. 10.7124/bc.000163

[B105] Tchantchou F. Goodfellow M. Li F. Ramsue L. Miller C. Puche A. (2021). Hyperhomocysteinemia-induced oxidative stress exacerbates cortical traumatic brain injury outcomes in rats. Cell. Mol. Neurobiol. 41 (3), 487–503. 10.1007/s10571-020-00866-7 32405706 PMC11448695

[B106] Vijayan M. Chinniah R. Ravi P. M. Sivanadham R. Mosses Joseph A. K. Vellaiappan N. A. (2016). MTHFR (C677T) CT genotype and CT-apoE3/3 genotypic combination predisposes the risk of ischemic stroke. Gene 591 (2), 465–470. 10.1016/j.gene.2016.06.062 27378745

[B107] Wei L. K. Au A. Menon S. Gan S. H. Griffiths L. R. (2015). Clinical relevance of MTHFR, eNOS, ACE, and ApoE gene polymorphisms and serum vitamin profile among Malay patients with ischemic stroke. J. Stroke Cerebrovasc. Dis. 24 (9), 2017–2025. 10.1016/j.jstrokecerebrovasdis.2015.04.011 26187788

[B108] Wenping S. Qi W. Mingquan S. (2003). Genetic polymorphisms of 5,10-methylenetetrahydrofolate reductase (MTHFR) in patients with cerebral infarction. Chin. J. Geriatr. Cardiovasc Cerebrovasc. D., 36–38.

[B109] Wu Y. Tomon M. Sumino K. (2001). Methylenetetrahydrofolate reductase gene polymorphism and ischemic stroke: Sex difference in Japanese. Kobe J. Med. Sci. 47 (6), 255–262. 11870335

[B110] Xi H. Zhang Y. Xu Y. Yang W. Y. Jiang X. Sha X. (2016). Caspase-1 inflammasome activation mediates homocysteine-induced pyrop-apoptosis in endothelial cells. Circ. Res. 118 (10), 1525–1539. 10.1161/CIRCRESAHA.116.308501 27006445 PMC4867131

[B111] Xin X. Y. Song Y. Y. Ma J. F. Fan C. N. Ding J. Q. Yang G. Y. (2009). Gene polymorphisms and risk of adult early-onset ischemic stroke: A meta-analysis. Thromb. Res. 124 (5), 619–624. 10.1016/j.thromres.2009.07.007 19660787

[B112] Xinliang Z. (1999). The relationship of polymorphisms of MTHFR gene and plasma homocysteine levels with stroke. Chin. J. Cardiol. 1, 60–62.

[B113] Yan S. Chengguo Z. Xueqiang H. (2006). Relationship of plasma homocysteine polymorphism in its enzyme genes and cerebral infarction in the elderly. J. Clin. Neurol., 22–24.

[B114] Yan S. Chengguo Z. Chijin S. Jinhuan C. (2003). Relationship between plasma HCY, polymorphism in MTHFR and cerebral thombosis. Henan J. Pract. Nerv. Dis. 3, 5–7.

[B115] Yanqun X. Lingli J. Qing L. Yinhua L. Qinghui Z. (2006). The relationship between the gene polymorphism of methylenetetrahydrofolate reductase and plasma homocysteine level with cerebrovascular disease. Lab. Med. 3, 201–204.

[B116] Yaqin Y. Bo L. Jieping S. Le Y. Liping Y. (2001). Study on the relationship between methylenetetrahydrofolate reductase gene polymorphism and acute cerebrovascular disease. J. N. BETHUNEUNIV.Med. Sci. 6, 623–625. 10.13481/j.1671-587x.2001.06.032

[B117] Yi F. Jianrong L. Peihua N. Yayun Y. Shengdi C. (2005). The relationship of plasma homocysteine levels and polymorphism in homocysteine metabolism related enzymes with brain stroke. Chin. J. G. eriat, 6, 413–417.

[B118] Youk J. An Y. Park S. Lee J. K. Ju Y. S. (2020). The genome-wide landscape of C: G > T: A polymorphism at the CpG contexts in the human population. BMC Genomics 21 (1), 270. 10.1186/s12864-020-6674-1 32228436 PMC7106825

[B119] Yue H. Wang Y. Zhang H. Liu J. (2010). Relationship between the plasma homocysteine levels and the polymorphisms of its metabolic enzymes and the brain infarction. Shanxi Med. J. 39 (02), 108–111.

[B120] Zak I. Sarecka-Hujar B. Kopyta I. Emich-Widera E. Marszal E. Wendorff J. (2009). The T allele of the 677C>T polymorphism of methylenetetrahydrofolate reductase gene is associated with an increased risk of ischemic stroke in Polish children. J. Child. Neurol. 24 (10), 1262–1267. 10.1177/0883073809333527 19805823

[B121] Zhang P. Guo X. (2012). Qibi, correlational study among the polymorphism of MTHFR gene, the plasma tHcy level and acute ischemic stroke in young adult. Chin. J. Stroke 7 (04), 271–277.

[B122] Zhang Y. Xie R. P. Shen Y. Fan D. S. (2008). Interaction between methylenetetrahydrofolate reductase C677T gene polymorphism and sleep duration on risk of stroke pathogenesis. Beijing Da Xue Xue Bao Yi Xue Ban. 40 (3), 262–269. 18560453

[B123] Zhang Y. Zhao Y. Gu B. (2016). Role of Hcy-related C677T gene mutation in hemorrhagic cerebral infarction. Chin. J. Geriatric Heart Brain Vessel Dis. 18 (06), 577–580.

[B124] Zheng Y. Z. Tong J. Do X. P. Pu X. Q. Zhou B. T. (2000). Prevalence of methylenetetrahydrofolate reductase C677T and its association with arterial and venous thrombosis in the Chinese population. Br. J. Haematol. 109 (4), 870–874. 10.1046/j.1365-2141.2000.02112.x 10929044

[B125] Zhou B.-S. Bu G. Y. Li M. Chang B. G. Zhou Y. P. (2014). Tagging SNPs in the MTHFR gene and risk of ischemic stroke in a Chinese population. Int. J. Mol. Sci. 15 (5), 8931–8940. 10.3390/ijms15058931 24853127 PMC4057767

