# Supplementary figures and images for: Association of methylenetetrahydrofolate reductase (MTHFR) rs1801133 (677C>T) gene polymorphism with ischemic stroke risk in different populations: An updated meta-analysis
Source: Front Genet. 2023 Jan 4;13:1021423. doi: 10.3389/fgene.2022.1021423 (PMC9845415; doi:10.3389/fgene.2022.1021423)

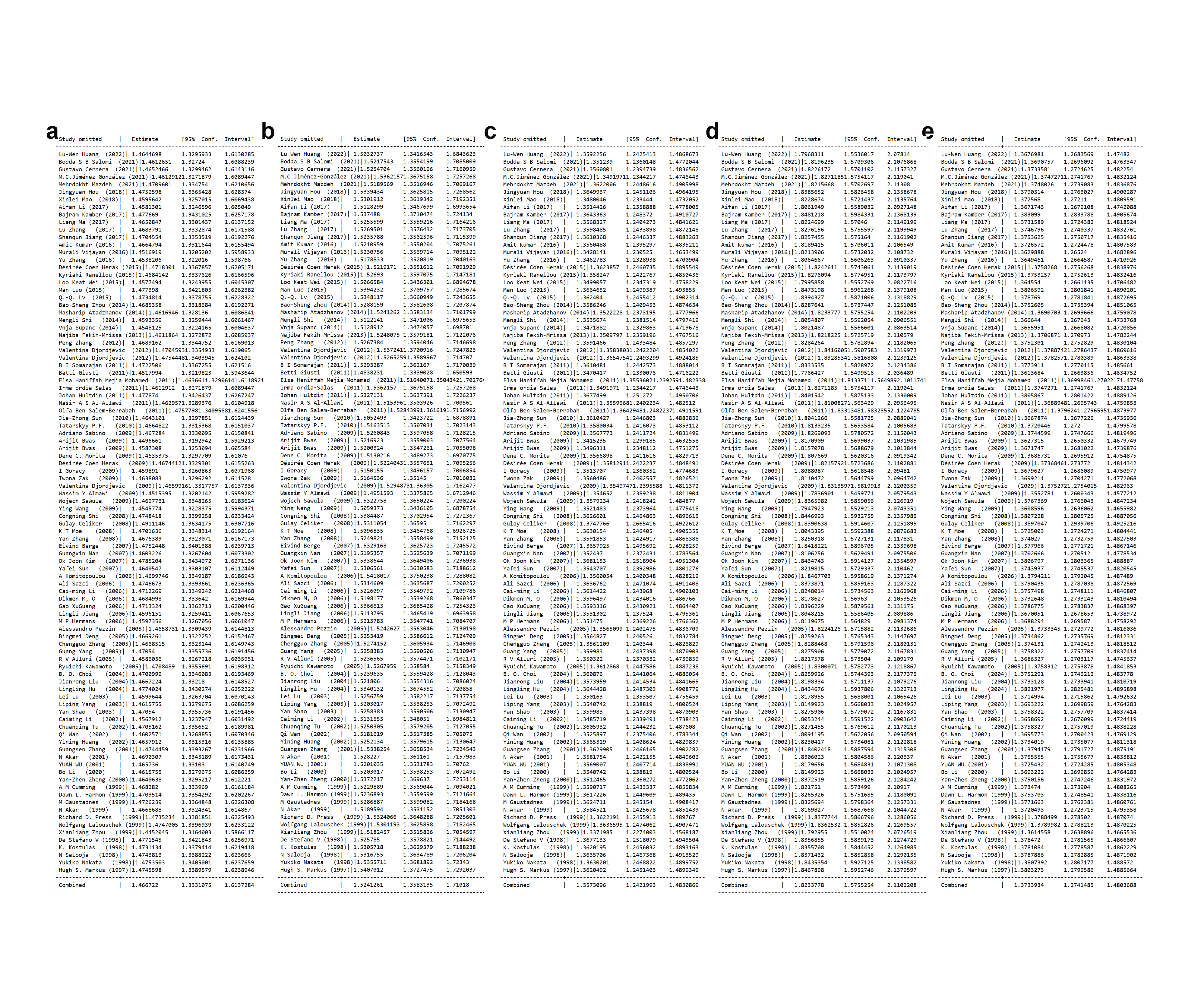

Supplement: Supplementary file 1 [file Image1.TIF]
